# From Detection to Diagnosis: An Advanced Transfer Learning Pipeline Using YOLO11 with Morphological Post-Processing for Brain Tumor Analysis for MRI Images

**DOI:** 10.3390/jimaging11080282

**Published:** 2025-08-21

**Authors:** Ikram Chourib

**Affiliations:** Independant Researcher, 75000 Paris, France; chourib.ikram@gmail.com

**Keywords:** brain tumor detection, computer-aided diagnosis, classification, deep learning, medical image, post-processing, transfer learning, tumor segmentation, YOLO

## Abstract

Accurate and timely detection of brain tumors from magnetic resonance imaging (MRI) scans is critical for improving patient outcomes and informing therapeutic decision-making. However, the complex heterogeneity of tumor morphology, scarcity of annotated medical data, and computational demands of deep learning models present substantial challenges for developing reliable automated diagnostic systems. In this study, we propose a robust and scalable deep learning framework for brain tumor detection and classification, built upon an enhanced YOLO-v11 architecture combined with a two-stage transfer learning strategy. The first stage involves training a base model on a large, diverse MRI dataset. Upon achieving a mean Average Precision (mAP) exceeding 90%, this model is designated as the Brain Tumor Detection Model (BTDM). In the second stage, the BTDM is fine-tuned on a structurally similar but smaller dataset to form Brain Tumor Detection and Segmentation (BTDS), effectively leveraging domain transfer to maintain performance despite limited data. The model is further optimized through domain-specific data augmentation—including geometric transformations—to improve generalization and robustness. Experimental evaluations on publicly available datasets show that the framework achieves high mAP@0.5 scores (up to 93.5% for the BTDM and 91% for BTDS) and consistently outperforms existing state-of-the-art methods across multiple tumor types, including glioma, meningioma, and pituitary tumors. In addition, a post-processing module enhances interpretability by generating segmentation masks and extracting clinically relevant metrics such as tumor size and severity level. These results underscore the potential of our approach as a high-performance, interpretable, and deployable clinical decision-support tool, contributing to the advancement of intelligent real-time neuro-oncological diagnostics.

## 1. Introduction

Brain cancer represents a critical global health challenge, responsible for numerous fatalities even in high-income countries. In the United States alone, approximately 20,000 deaths annually are attributed to brain tumors, underscoring the urgent need for improved diagnostic and treatment strategies [1]. Brain cancer is characterized by uncontrolled cellular proliferation, resulting from disruptions in normal cell-regulation processes. Typically, the human body maintains cell homeostasis through systematic cell division, replacing aged or damaged cells promptly. However, this finely regulated mechanism can malfunction, allowing abnormal cells to multiply unchecked, leading to the formation of tumor masses. Brain tumors can be categorized into primary tumors, originating directly within the brain tissue, and secondary tumors, which arise elsewhere in the body and subsequently metastasize to the brain [2,3,4].

Tumors are further classified as malignant or benign based on their biological behavior [5]. Malignant tumors infiltrate surrounding tissues and are capable of metastasizing to distant body regions, complicating treatment and significantly affecting prognosis. In contrast, benign tumors generally remain localized, do not invade adjacent tissues, and rarely recur following surgical removal. Despite their non-invasive nature, benign tumors can grow substantially large, potentially causing severe neurological symptoms and life-threatening complications due to increased intracranial pressure or compromised brain function.

Specific brain tumor types, including meningiomas, gliomas, and pituitary adenomas, are distinguished based on their cell of origin and clinical manifestations. Meningiomas, comprising approximately 36.1% of primary brain tumors, typically develop from the meninges, the protective membranes surrounding the brain and spinal cord. Although usually benign, meningiomas can become life-threatening due to their strategic anatomical positioning, leading to severe clinical symptoms such as seizures and visual impairment [6,7]. Gliomas arise from glial cells, integral components supporting neuronal function. These tumors exhibit considerable heterogeneity in malignancy, growth rate, and clinical outcomes, posing substantial challenges for effective management. Pituitary tumors originate near the pituitary gland at the base of the skull, influencing diverse physiological processes due to hormonal dysregulation.

Advancements in imaging technologies have significantly improved the detection and characterization of brain tumors, enabling earlier intervention and more personalized therapeutic strategies. Among the available modalities—such as computed tomography (CT), ultrasound, magnetoencephalography (MEG), electroencephalography (EEG), and X-ray—magnetic resonance imaging (MRI) remains the gold standard due to its superior soft tissue contrast, which allows the precise identification of brain lesions [8,9]. Nevertheless, interpreting MRI scans requires specialized expertise that is often unavailable in smaller healthcare settings, leading to delayed diagnoses and potentially compromised patient outcomes.

In recent years, deep learning—particularly convolutional neural networks (CNNs)—has demonstrated remarkable potential in medical imaging, achieving high performance in tumor detection, classification, and segmentation tasks [10,11,12,13,14]. However, the existing CNN-based computer-aided diagnosis (CAD) systems present important limitations. Many state-of-the-art models demand substantial computational resources, which hinders their deployment in standard clinical environments. Lightweight CNN architectures improve computational efficiency but often sacrifice detection precision, especially for small or complex lesions [15]. Segmentation-based methods provide detailed tumor delineation but are computationally expensive and difficult to implement on conventional hardware. Furthermore, while modern object detection frameworks such as SSD, R-CNN, and Faster R-CNN [16,17,18,19,20] have improved efficiency, their clinical applicability is constrained by trade-offs between accuracy, inference speed, and hardware compatibility. Even high-performing YOLO variants [21]—despite balancing speed and accuracy—have rarely been optimized for the unique challenges of brain MRI, including heterogeneous tumor morphology, variable acquisition protocols, and limited annotated data. Notably, despite rapid algorithmic advances, the number of deep learning-based brain image analysis software solutions that have undergone successful clinical validation remains limited, with only a small subset (fewer than 10 systems to date) approved or cleared for clinical use worldwide [22].

To address these gaps, this study proposes a clinically relevant end-to-end deep learning framework for brain tumor detection, classification, and segmentation from MRI scans. Our approach introduces a two-stage transfer learning strategy in which a base model is first trained on a large, diverse dataset and then fine-tuned on a smaller domain-specific dataset to enhance generalization with limited data. The framework integrates an optimized YOLOv11-based detector enhanced with domain-specific augmentation techniques—including horizontal flipping, mosaic, and cutmix—to achieve both high detection accuracy and real-time inference performance on standard clinical hardware. Additionally, a post-processing module provides tumor size measurements and severity estimations, bridging the gap between raw model outputs and radiological decision-making. The pipeline is designed to be modular, scalable, and easily deployable in low-resource settings.

The main contributions of this work can be summarized as follows:

- A two-stage transfer learning framework tailored for brain tumor MRI analysis, enabling robust detection and segmentation with limited domain-specific data.
- The integration of an optimized YOLOv11 detector with customized augmentation strategies for improved robustness and real-time performance.
- The development of a clinically oriented post-processing module that provides interpretable quantitative outputs to support radiological review.
- A scalable and resource-efficient architecture suitable for deployment in standard healthcare environments, even with limited computational capacity.

The remainder of this paper is organized as follows: Section 2 reviews the related literature and highlights existing research gaps; Section 3 describes the proposed methodological framework; Section 4 presents the experimental results and performance analysis; and Section 5 concludes the paper with a summary of the findings and considers future research directions.

## 2. Related Works

The integration of advanced deep learning techniques, particularly You-Only-Look-Once (YOLO) architectures, has significantly enhanced tumor detection and segmentation capabilities across diverse medical imaging modalities. YOLO frameworks are particularly valued for their rapid real-time analysis and precise object localization, enabling efficient diagnosis and decision-making.

George et al. [21] utilized YOLO-based deep learning models specifically tailored for the real-time identification and precise localization of lung nodules in low-dose CT scans, achieving significant improvements in detection speed and accuracy. However, their model was limited to lung CT images, and its generalizability to other modalities, such as MRI, remains untested. Hossain et al. [23] developed a portable microwave imaging system coupled with a YOLO-based deep learning model, effectively classifying and detecting brain abnormalities. Their work highlights the potential for deploying YOLO models in portable diagnostic devices. Nevertheless, the diagnostic accuracy may be constrained by the lower spatial resolution of microwave imaging compared to standard radiological imaging. Shelatkar and Bansal [24] proposed a lightweight YOLO model combined with a fine-tuning approach to efficiently diagnose brain tumors from MRI images. The model achieved computational efficiency, making it suitable for real-time application. A limitation noted was the reduced performance on complex cases with overlapping or small tumor regions. Talukder et al. [25] introduced an innovative approach combining image-reconstruction methods with deep learning fine-tuning to enhance classification accuracy in MRI-based brain tumor categorization. Although highly effective, the method required considerable preprocessing and model tuning, potentially limiting its adaptability to diverse datasets without retraining.

Salman et al. [26] explored automated detection methodologies by integrating hybrid image-processing techniques with YOLO-based architectures. This approach significantly improved detection accuracy. However, the hybrid pipeline introduced additional complexity and computational overhead, reducing its suitability for lightweight or portable applications. Havaei et al. [27] developed comprehensive deep neural networks for precise brain tumor segmentation in MRI imaging. Their framework provided highly accurate segmentation, yet it required large annotated datasets and extensive computational resources, which may hinder clinical deployment in low-resource settings. Ben Brahim et al. [28] introduced a deep convolutional neural network (CNN) optimized for brain tumor detection from MRI images, offering efficient and reliable performance. The study, however, did not benchmark the model against other state-of-the-art detectors such as YOLO, leaving its comparative performance unclear.

Adhikari et al. [29] leveraged parameter-efficient fine-tuning strategies to improve CNN baselines in brain tumor segmentation, particularly focusing on the sub-Saharan Africa adult glioma dataset. While their model achieved notable performance enhancements, its regional specificity may limit generalization to global datasets without adaptation. Mabray et al. [30] reviewed modern brain tumor imaging techniques, especially advancements in MRI. Their review provides a strong foundation for imaging-based diagnosis but lacks integration with emerging AI-based analysis techniques that have become critical in recent years.

While not directly related to medical imaging, Cengil and Çınar [31] illustrated the versatility of YOLOv5 in detecting poisonous mushrooms. This study emphasized YOLO’s adaptability; however, it provides limited insights into the challenges of applying YOLO in medical domains, where image variability and annotation scarcity are prominent issues.

In this context, Table 1 summarizes representative studies employing YOLO-based and deep learning methods for tumor detection, highlighting their modalities, key contributions, and limitations. These detailed insights emphasize both the advancements and remaining challenges in the use of deep learning and YOLO-based approaches for medical image analysis. Addressing issues such as domain generalization, computational efficiency, and clinical integration remains crucial for real-world deployment.

**Table 1:** Comprehensive overview of YOLO-based and deep learning studies for tumor detection.

Study	Modality	Approach	Key Contribution	Result Highlights	Limitations
George et al. (2018) [21]	CT scans	YOLO-based deep learning	Real-time lung nodule detection and localization	Rapid and accurate clinical detection	Limited to CT lung scans; lacks generalization to MRI
Hossain et al. (2022) [23]	Microwave imaging	YOLO-based deep learning model	Portable system for classifying brain abnormalities	High accuracy, enhanced portability	Lower resolution limits diagnostic detail
Shelatkar & Bansal (2023) [24]	MRI	Lightweight YOLO model with fine-tuning	Efficient and reliable tumor diagnosis	Effective for resource-limited settings	Reduced accuracy on complex or small tumors
Talukder et al. (2023) [25]	MRI	Reconstruction combined with fine-tuning	Improved accuracy in tumor categorization	Substantial performance enhancement	Requires significant preprocessing and tuning
Salman et al. (2022) [26]	MRI	Hybrid image processing integrated with YOLO	Enhanced automated tumor detection reliability	Superior performance over traditional methods	Increased pipeline complexity and computational load
Havaei et al. (2017) [27]	MRI	Deep neural networks	Accurate brain tumor segmentation	Precise delineation of tumor boundaries	High data and compute requirements
Ben Brahim et al. (2024) [28]	MRI	Deep CNN model	Efficient and reliable tumor detection	Rapid identification and high reliability	Lacks comparative benchmarking with YOLO or other detectors
Adhikari et al. (2024) [29]	MRI	Parameter-efficient fine-tuning	Improved segmentation tailored to regional datasets	Notable enhancement in local diagnostics	Regional focus may limit global generalizability
Mabray et al. (2015) [30]	MRI	Modern MRI imaging review	Advanced imaging techniques for tumor characterization	Enhanced diagnosis and treatment planning	Lacks integration with modern AI-based diagnostic tools
Cengil and Çınar (2021) [31]	Image detection	YOLOv5	Demonstration of YOLO versatility across object detection tasks	Broadened applicability and high accuracy	Not tailored to medical imaging domain complexities

## 3. Proposed Methodology

**Figure 1:**
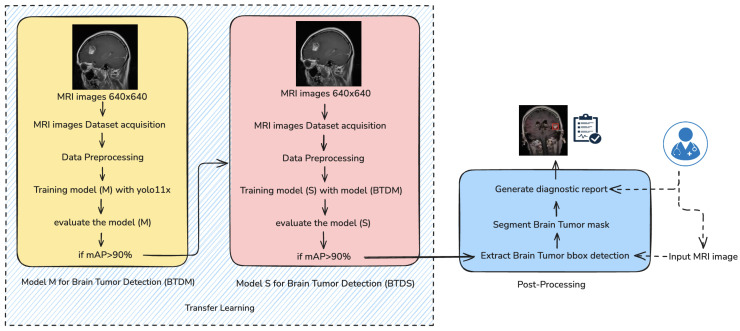
The proposed methodology based on transfer learning (BTDM–BTDS). The brain MRI scans are first input into the detection or segmentation model. The resulting outputs are then processed by the post-processing module to generate clinically relevant indicators (tumor size, shape, and severity). These outputs are subsequently reviewed by the physician. The physician does not directly insert MRI images into the post-processing block.

The proposed brain tumor detection and segmentation framework follows a multistage deep learning pipeline, as shown in Figure 1, combining transfer learning, precise preprocessing, and advanced post-processing to deliver accurate diagnostic outputs. The methodology comprises five key components: data collection, data preprocessing, model training, model evaluation, and post-processing.

As depicted in Figure 1, the training process follows a two-stage architecture. In the initial stage, a base detection model M is trained using the preprocessed dataset and evaluated based on the mean Average Precision (mAP) metric. If model M achieves an mAP greater than 90%, it is designated as the Brain Tumor Detection Model (BTDM). This model is subsequently transferred to a second training phase, where it serves as the initialization for model S, trained on a distinct but structurally similar dataset using transfer learning principles. The final output model S, denoted as BTDS (Brain Tumor Detection System), is used in a post-processing pipeline to extract bounding boxes, generate segmentation masks, and produce a diagnostic report.

### 3.1. Model-Training Strategy

The proposed brain tumor detection framework is built upon YOLO-v11x, a state-of-the-art high-throughput object detection architecture optimized for both accuracy and inference speed. In the following sections, we detail the internal structure of YOLOv11 and explain how this model serves as the core of our two-phase learning strategy. This includes the initial training stage of a base model (BTDM), followed by a transfer learning phase to obtain a domain-specialized model (BTDS). Together, these stages form a robust and efficient detection pipeline tailored for brain MRI tumor analysis.

#### 3.1.1. YOLO Network Architecture

**Figure 2:**
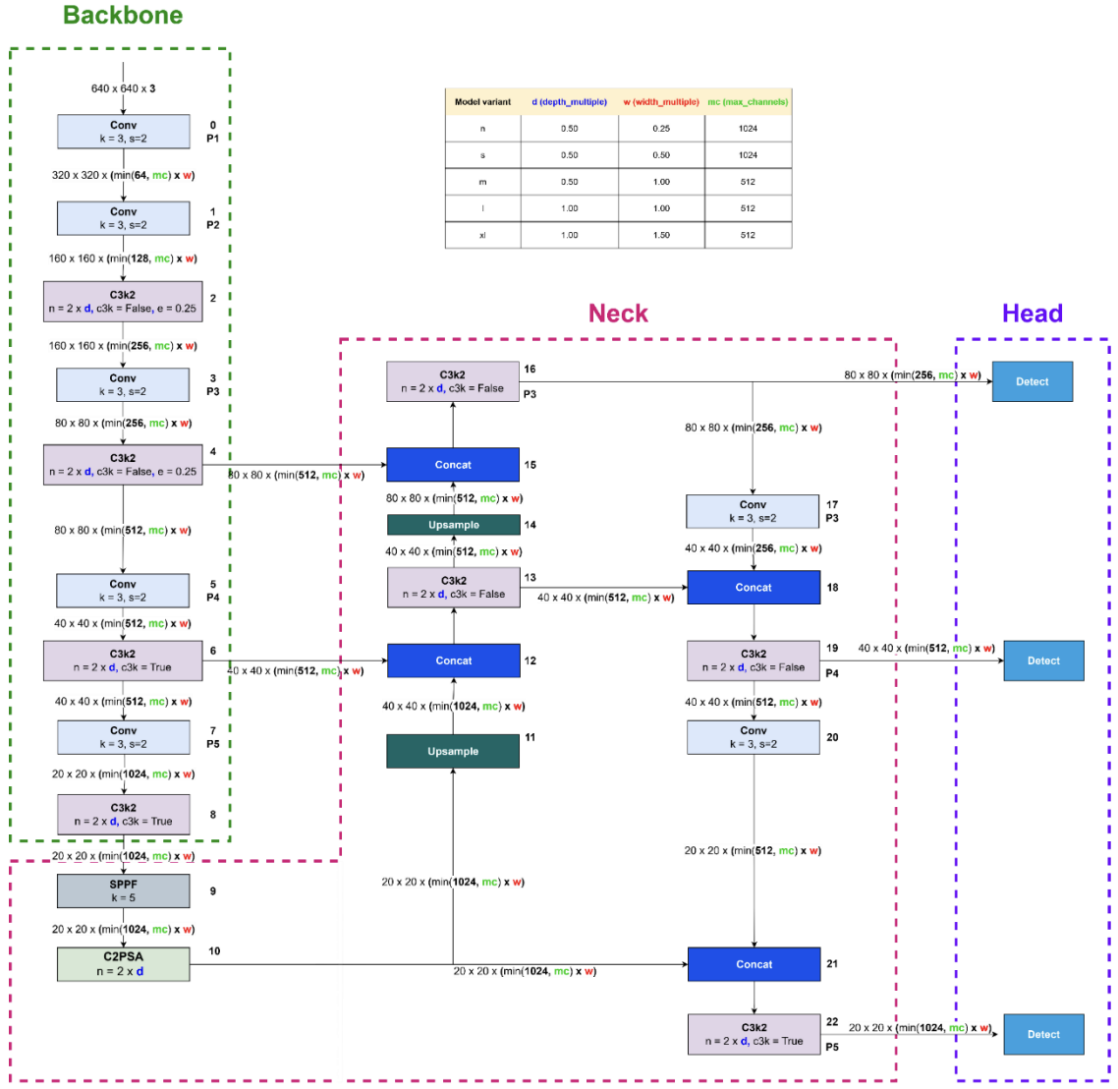
YOLO11 architecture (adapted from [34]). Block colors indicate operations: Green dashed: Backbone (feature extraction). Pink dashed: Neck (multi-scale feature fusion). Violet dashed: Head (final detection). Light gray—Conv: standard convolution operation used to extract local features. Light violet—C3k2: residual block combining two convolutions to enhance feature representation while reducing complexity. Bluish gray—SPPF: Spatial Pyramid Pooling Fast, aggregates information at multiple receptive fields with low computational cost. Steel gray—C2PSA: Cross Stage Partial Attention, improves feature flow by integrating partial attention mechanisms. Dark blue—Concat: concatenation of features from different layers to enrich multi-level representations. Light blue—Upsample: spatial upsampling to merge feature maps of different resolutions. Sky blue—Detect: multi-scale detection heads producing final predictions (bounding boxes, classes, and confidence scores).

The architecture illustrated in Figure 2 is derived from the configuration file yolo11.yaml, located in the ultralytics/cfg/models/11 directory [32]. Similar to YOLOv8, the YOLOv11 architecture is parameterized by three key variables: depth_multiple, width_multiple, and max_channels. These parameters govern the network’s structural complexity and channel capacity. Specifically, while width_multiple and max_channels determine the number of output channels, the depth_multiple variable specifies the number of C3k bottleneck blocks within the C3k2 modules and the PSA blocks in the C2PSA component—an architectural distinction from previous YOLO versions.

The stem structure of YOLOv11 remains identical to that of YOLOv8. However, in the stage component, YOLOv11 introduces a novel C3k2 block, which is a lightweight and more parameter-efficient extension of the C2f block found in YOLOv8 and YOLOv10. The behavior of C3k2 is controlled by the c3k flag: when c3k=True, the C3k module is used; otherwise, the architecture defaults to a bottleneck design, as in C2f. Importantly, for the medium (m), large (l), and extra-large (x) variants of YOLOv11, c3k is always set to True. This architectural detail is undocumented in the official YOLOv11 materials [33] but was identified through careful inspection of the source code—highlighting the importance of source-level examination for a complete understanding of the model.

YOLOv11’s backbone consists of eight stages, specifically blocks 2, 4, 6, 8, 13, 16, 19, and 22. Downsampling is performed using convolutional layers with a kernel size of 3 and a stride of 2, reducing spatial resolution by 50% at each step.

The neck of YOLOv11 integrates an SPPF (Spatial Pyramid Pooling-Fast) module—similar to that in YOLOv8—followed by a C2PSA block. Inspired by the PSA module from YOLOv10, C2PSA incorporates a self-attention mechanism that enhances feature representation by capturing global contextual dependencies. In YOLOv11, the C2PSA block is positioned immediately after stage 4, where feature maps are at their lowest resolution. As in YOLOv8 and YOLOv10, the neck also includes concat and nearest-neighbor upsample blocks.

Finally, the head architecture of YOLOv11 mirrors that of YOLOv10 in both structure and functionality, maintaining consistency for object detection outputs.

#### 3.1.2. Learning Process

To maximize its applicability in the neuroimaging domain, the training process is organized into two distinct but complementary phases:

- Stage I—Initial Training of Model M (BTDM): In the first stage, a base detection model—referred to as Model M—was trained from scratch using a comprehensive dataset of annotated brain magnetic resonance imaging scans. The dataset encompassed a wide range of tumor types and anatomical variations, enabling the model to learn robust and discriminative features for tumor localization. The training objective was to minimize a composite loss function comprising localization, classification, and objectness confidence terms. Upon convergence, the model was evaluated on a held-out validation set using mean Average Precision (mAP) as the primary performance metric. If mAP ≥ 90% at IoU = 0.5, the model was designated as the Brain Tumor Detection Model (BTDM) and considered suitable for knowledge transfer.
- Stage II—Transfer Learning to Model S (BTDS): To enhance generalization and reduce training time on smaller or domain-specific datasets, a transfer learning approach was employed. The previously trained BTDM was used to initialize a secondary detection model—Model S—which was then fine-tuned on a different but structurally consistent dataset. This second dataset maintained similar annotation formats and imaging protocols, allowing for seamless weight transfer while preserving domain-specific priors. Fine-tuning was performed with a reduced learning rate and early stopping mechanisms to prevent overfitting. Once Model S surpassed the same performance threshold (mAP ≥ 90%), it was finalized as Brain Tumor Detection and Segmentation (BTDS) and integrated into the downstream inference pipeline for clinical post-processing and reporting (see Figure 1).

### 3.2. Advanced Post-Processing Pipeline for Clinically Informed Brain Tumor Analysis

Following the initial detection of brain tumors by Brain Tumor Detection and Segmentation (BTDS), an advanced and modular post-processing pipeline is systematically deployed to transform raw model predictions into anatomically precise and clinically actionable diagnostic insights. This multistage framework is designed not merely as a technical enhancement but as a translational bridge between data-driven inference and clinical applicability. By incorporating spatial refinement techniques, morphometric analysis, and semantic interpretation, the pipeline ensures that outputs are not only accurate but also interpretable and aligned with diagnostic decision-making processes. Furthermore, its architecture supports seamless integration within established radiological workflows, including Picture Archiving and Communication Systems (PACSs) and Radiology Information Systems (RISs), thereby facilitating real-time decision support, surgical planning, and longitudinal patient monitoring in routine clinical settings.

### 3.3. Spatial Localization via Bounding Box Extraction

Initially, BTDS provides coarse spatial localization through bounding boxes (BBOXs), each associated with tumor class predictions and confidence scores. Formally, a bounding box *B* is defined by

(1) B=(x,y,w,h,c,p)

where (x,y) denotes the top-left coordinate of the box, *w* and *h* its width and height, respectively, *c* the tumor class label, and *p* the associated confidence score. These bounding boxes act as spatial priors, drastically reducing computational complexity for subsequent pixel-level refinement.

#### 3.3.1. Anatomically Precise Segmentation Refinement

To achieve precise tumor delineation, each bounding box region undergoes targeted segmentation refinement using the grayscale intensities I(x,y) within the box region. The segmentation refinement comprises three key steps:

Adaptive Thresholding with Otsu’s Method:

Within the bounding box region R⊆I, an adaptive binarization is performed using Otsu’s threshold $\tau_{\mathrm{otsu}}$:

(2) $$\tau_{\mathrm{otsu}}=\arg\max_{\tau }\;\sigma_{b}^{2}\left(\tau \right)$$

where $\sigma_{b}^{2}$ is the between-class variance defined as

(3) $$\sigma_{b}^{2}\left(T\right)=w_{1}\left(T\right)\;w_{2}\left(T\right){\left[\mu_{1}\left(T\right)-\mu_{2}\left(T\right)\right]}^{2}$$

Here, $w_{1}\left(T\right)$ and $w_{2}\left(T\right)$ are class probabilities, and $\mu_{1}\left(T\right)$ and $\mu_{2}\left(T\right)$ denote class means. This step yields a preliminary binary mask $M_{\mathrm{otsu}}\left(x,y\right)$:

(4) $$M_{\mathrm{otsu}}\left(x,y\right)=\left\{\begin{array}{cc} 1, & I\left(x,y\right)>T_{\mathrm{otsu}}\\ 0, & \mathrm{otherwise} \end{array}\right.$$

Hole Filling for Topological Continuity:

To ensure anatomical plausibility, internal holes within the preliminary mask $M_{\mathrm{otsu}}$ are filled using morphological operations. Given the external contours *C*, the filled mask $M_{\mathrm{filled}}\left(x,y\right)$ is computed as follows:

(5) $$M_{\mathrm{filled}}\left(x,y\right)=\mathrm{fillContours}\left(C\right)$$

which provides spatial continuity and robustness against artifacts in MRI intensity variations.

Contour Smoothing via Morphological Operations:

To refine the mask borders, we sequentially apply morphological opening (∘) and closing (•) operations. Here, ∘ denotes morphological opening (erosion followed by dilation) and • denotes morphological closing (dilation followed by erosion), both with structuring element *S* (e.g., a 3×3 square). This smoothing procedure removes noise and small spurious regions, leading to anatomically more realistic and continuous tumor boundaries:

(6) $$M_{\mathrm{smooth}}=\left(M_{\mathrm{filled}}\circ S\right)•S$$

Clinical Visualization (Red Overlay):

For clinical interpretability, the refined mask $M_{\mathrm{smooth}}$ is overlaid onto the original MRI slice using color-coding (commonly red):

(7) $$I_{\mathrm{overlay}}\left(x,y\right)=\left\{\begin{array}{cc} \mathrm{Red}, & M_{\mathrm{smooth}}\left(x,y\right)=1\\ I(x,y), & \mathrm{otherwise} \end{array}\right.$$

#### 3.3.2. Morphometric and Quantitative Feature Extraction

Subsequent to refined segmentation, quantitative morphological features are extracted. The linear dimensions (length *L* and width *W*) are directly inferred from the bounding box dimensions:

(8) $$L_{\mathrm{cm}}=h\times f_{\mathrm{cm}/\mathrm{pixel}},\;W_{\mathrm{cm}}=w\times f_{\mathrm{cm}/\mathrm{pixel}}$$

where $f_{\mathrm{cm}/\mathrm{pixel}}$ is the pixel-to-centimeter scaling factor calibrated from MRI metadata. Tumor area *A* and perimeter *P* are directly calculated from the segmentation mask:

(9) $$A=\sum_{\left(x,y\right)}M_{\mathrm{smooth}}\left(x,y\right)\times f_{\mathrm{cm}/\mathrm{pixel}}^{2},\;P=\sum_{\mathrm{boundary}\;\mathrm{pixels}}f_{\mathrm{cm}/\mathrm{pixel}}$$

Shape descriptors such as eccentricity and compactness further characterize tumor morphology and potential malignancy indicators.

#### 3.3.3. Quantitative Severity Index Estimation

A quantitative malignancy severity index *S* integrates size, location, and morphological complexity according to WHO CNS tumor classification guidelines:

(10) $$S=f_{\mathrm{severity}}\left(A,L,W,\mathrm{location},\mathrm{shape}\;\mathrm{complexity}\right)$$

Specifically, tumor size categories are defined as

- Small: L<2cm.
- Medium: 2≤L≤4cm.
- Large: L>4cm.

Additional complexity factors (e.g., irregular borders) are incorporated to accurately reflect clinical severity.

### 3.4. Automated Structured Diagnostic Report

Finally, a structured diagnostic report encapsulates tumor classification, morphometric dimensions, computed severity index, and associated clinical interpretations. The report is formatted to ensure direct integration into Picture Archiving and Communication Systems (PACSs) or Radiology Information Systems (RISs), facilitating immediate clinical action.

### 3.5. Theoretical Significance and Clinical Relevance

This integrated computational framework uniquely combines deep learning-driven tumor detection, classical image-processing refinement, and quantitative morphological characterization, thereby establishing a robust and reproducible clinical tool. The proposed pipeline is modular, adaptable, and directly aligned with diagnostic radiology protocols, enabling efficient clinical workflows and improved patient-care outcomes.

## 4. Experimental Results

This section presents a comprehensive evaluation of the proposed models through key performance metrics, including loss convergence, precision, recall, and mAP@50. The analysis highlights the diagnostic reliability and robustness of the system. A confusion matrix further illustrates class-wise performance. These results provide critical insights into the clinical applicability and limitations of the approach.

### 4.1. Data Collection

To construct robust and generalizable models for brain tumor detection and segmentation, we curated a high-quality dataset of annotated magnetic resonance imaging (MRI) scans sourced from publicly available neuroimaging repositories and anonymized hospital archives. This collection strategy ensured diversity in tumor morphology, acquisition protocols, and MRI modalities. Each MRI slice was manually annotated, with axis-aligned bounding boxes provided for detection tasks and pixel-level segmentation masks for precise delineation of tumor regions when required. Only images containing verified tumor annotations were retained, thereby eliminating non-informative background slices and maintaining a focused learning paradigm. The dataset was stratified into training and validation subsets using a 90:10 split ratio to preserve class balance across tumor types. For the detection model (BTDM), the training set consisted of 7929 images, with 886 images reserved for validation, covering pituitary, glioma, and meningioma tumors. The corresponding data sources are publicly accessible via Roboflow Dataset 1 (https://universe.roboflow.com/final-eyj5r/brain-mri-pqpcm, accessed on 20 May 2025) and Dataset 2 (https://universe.roboflow.com/my-thesis-zy14w/mergechngbvj, accessed on 20 May 2025). For the segmentation model (BTDS), 1183 images were used for training and 253 for validation, with tumor categories matching those in the detection dataset; this dataset is available via Roboflow Dataset 3 (https://universe.roboflow.com/lll-czafp/project2-blspd/dataset/2, accessed on 20 May 2025).

### 4.2. Data Preprocessing

A comprehensive and methodically designed data preprocessing pipeline was implemented to improve the generalization capabilities of the model, mitigate overfitting, and ensure high fidelity between the input data and the corresponding ground-truth annotations. This pipeline was critical in establishing a consistent and robust foundation for training and comprised the following key components:

- Annotation Quality Assurance and Conversion: To ensure spatial fidelity, all the bounding box annotations, originally provided by the dataset creators, were manually inspected and, when necessary, corrected by Dr. Aya Sahbia Chourib, a resident at Mongi Slim Hospital in Tunisia, particularly focusing on alignment with tumor margins in MRI slices. This manual verification step was essential to eliminate inaccurate or misaligned labels, which could otherwise propagate error during training. Subsequently, annotations originally provided in COCO JSON format were programmatically converted into YOLO-compatible text (.txt) label files, facilitating seamless integration into the YOLOv8 training pipeline.
- Annotation Filtering: Non-informative MRI slices—specifically those devoid of tumor annotations—were excluded from the training set. This filtering step mitigated the risk of introducing class imbalance and reduced the likelihood of the model learning misleading background representations under supervised learning assumptions.
- Data Augmentation: To enhance the robustness and generalization capability of the model across varying anatomical contexts and imaging conditions, an extensive data augmentation strategy was employed. These augmentations aim to increase the diversity of training samples without requiring additional annotated data, thus mitigating overfitting and improving the model’s resilience to unseen data. These techniques simulate different spatial configurations and orientations of tumors, which is particularly useful given the variability in MRI acquisition planes and patient positioning [35,36]. Thus, the augmentations can be categorized as follows:
  - Horizontal Flipping: Implemented with ‘*p* = 0.5’ (probability), corresponding to random mirroring of the image along the vertical axis. This operation preserves anatomical plausibility while exposing the model to mirrored tumor instances, thereby improving its left–right invariance.
  - Vertical Flipping (flipud): Vertical axis mirroring is less common in clinical practice implemented with ‘*p* = 0.1’ but was included with controlled probability to simulate orientation variability in data sources.
  - Random Translation and Scaling: Applied with ‘translate = (0.1, 0.1)’ and ‘scale = (0.8, 1.2)’. Spatial shifting and resizing of tumor regions help the model to generalize across positional and size variations. Bounding box coordinates were updated accordingly to maintain annotation accuracy.
  - Mosaic Augmentation: Introduced in YOLOv4 [37], mosaic augmentation stitches four images into one by randomly cropping and combining them, implemented with ‘mosaic = 0.9’ probability. This increases the apparent object density, introduces varied contexts within a single training sample, and helps the model learn to detect tumors in cluttered or multi-lesion scenarios.
  - Copy–Paste Augmentation: Applied with ‘*p* = 0.4’. This technique involves extracting tumor regions from one image and pasting them into another background image. It is particularly effective for rare classes or small tumors, allowing for synthetic enrichment of underrepresented tumor types or anatomical zones without altering overall image realism.
  - MixUp Blending: Implemented with ‘mixup_alpha = 0.2’ and applied with probability ‘*p* = 0.1’, where two images and their corresponding labels are linearly combined using a convex combination, simulating soft transitions and occlusions. This regularization technique encourages the model to behave linearly between samples and reduces overconfidence on individual predictions.
- Implementation Details: All augmentations were applied online during the data-loading phase using a probabilistic scheduler, ensuring stochastic variability at each training epoch. Care was taken to maintain anatomical consistency and valid bounding box mappings post-transformation. Augmentation parameters (e.g., scale factors, translation offsets, and blending ratios) were empirically tuned based on model performance on a validation subset.
- Optimization Strategy: Training was carried out using stochastic gradient descent (SGD), a widely adopted optimizer in large-scale object detection tasks due to its convergence stability and efficiency. We employed dynamic learning rate scheduling, coupled with momentum tuning, to accelerate convergence and escape shallow local minima. These hyperparameters were tuned empirically to achieve optimal trade-offs between training stability and generalization.

### 4.3. Preliminary Experimental Investigation

All experiments were conducted on the Google Colab platform using a high-performance NVIDIA A100 GPU to ensure efficient training. The implementation was carried out in Python 3.10 using the Ultralytics YOLOv11 framework with the following main dependencies (minimum versions in parentheses): numpy (1.23.0), matplotlib (3.3.0), opencv-python (4.6.0), pillow (7.1.2), pyyaml (5.3.1), requests (2.23.0), scipy (1.4.1), torch (1.8.0), torchvision (0.9.0), tqdm (4.64.0), psutil, py-cpuinfo, pandas (1.1.4), and ultralytics-thop (2.0.0) for the computation of FLOPs.

Both models were optimized using stochastic gradient descent (SGD) with a learning rate of 0.001, weight decay of 0.0005, and momentum of 0.937. To improve generalization and mitigate overfitting, several regularization and augmentation strategies were employed, including dropout (0.4), mosaic (0.9), mixup (0.1), cutmix (0.3), and copy–paste (0.4). Training was performed for 100 epochs, with early stopping applied using a patience of 15 epochs to ensure stable convergence.

The datasets described in Section 4.1 were split into 90% for training and 10% for validation, with identical optimization and augmentation configurations maintained across both experiments to enable a fair performance comparison between detection and segmentation tasks.

To ensure that the reported results are robust and not influenced by accidental factors—such as favorable random initialization or biased dataset splits—all experiments were carried out in a controlled computational environment and repeated five times with different random seeds. For each run, the same training–validation split, optimization settings, and data augmentation pipeline were maintained to guarantee comparability. Final performance metrics, including mAP@0.5, precision, and recall, are reported as the mean and standard deviation across all runs. This multi-run evaluation strategy minimizes the impact of stochastic variations and confirms both the stability and reproducibility of the proposed framework.

### 4.4. Evaluation Metrics

To rigorously evaluate the performance of our tumor detection framework, we adopted a comprehensive set of evaluation metrics, including precision, recall, mean Average Precision (mAP), and individual loss components [38,39]. Collectively, these metrics provide a holistic assessment of the system by capturing classification accuracy, spatial localization precision, and the overall robustness and convergence behavior of the detection pipeline.

Precision, Recall, and F1-Score

Precision quantifies the proportion of correctly predicted tumor instances (true positives, *TPs* ) among all predicted positives (*TPs* + *FPs*) and is expressed as

(11) $$\mathrm{Precision}=\frac{\mathrm{TP}}{\mathrm{TP}+\mathrm{FP}}$$

where *TP* = true positive and *FP* = false positive. Recall, on the other hand, evaluates the model’s sensitivity by computing the ratio of true-positive detections to all actual tumor instances (*TP* + *FN*):

(12) $$\mathrm{Recall}=\frac{\mathrm{TP}}{\mathrm{TP}+\mathrm{FN}}$$

where *TP* = true positive and *FN* = false negative.

The F1-score combines precision and recall into a single metric by taking their harmonic mean, providing a balanced measure when both false positives and false negatives are important:

(13) $$F1\text{-}\mathrm{score}=2\times \frac{\mathrm{Precision}\times \mathrm{Recall}}{\mathrm{Precision}+\mathrm{Recall}}$$

Accuracy

Accuracy measures the proportion of correctly classified instances (both positive and negative) among all samples and is expressed as

(14) $$\mathrm{Accuracy}=\frac{\mathrm{TP}+\mathrm{TN}}{\mathrm{TP}+\mathrm{TN}+\mathrm{FP}+\mathrm{FN}}$$

where TP = true positive, TN = true negative, FP = false positive, and FN = false negative.

The Matthews Correlation Coefficient (MCC)

The Matthews Correlation Coefficient (MCC) is a balanced metric that takes into account true and false positives and negatives, and is particularly useful for evaluating classification performance on imbalanced datasets. It is defined as

(15) $$\mathrm{MCC}=\frac{\mathrm{TP}\times \mathrm{TN}-\mathrm{FP}\times \mathrm{FN}}{\sqrt{\left(\mathrm{TP}+\mathrm{FP})\;(\mathrm{TP}+\mathrm{FN})\;(\mathrm{TN}+\mathrm{FP})\;(\mathrm{TN}+\mathrm{FN}\right)}}$$

The *MCC* ranges from −1 (perfect disagreement) to +1 (perfect agreement), with 0 indicating random prediction.

Mean Average Precision (mAP)

To assess the detection performance across classes and thresholds, we used the mean Average Precision (mAP). Specifically, mAP@0.5 measures the Average Precision (AP) at an Intersection over Union (IoU) threshold of 0.5. Further, mAP@0.5:0.95 represents the mean AP averaged over ten IoU thresholds ranging from 0.5 to 0.95 with a step size of 0.05. The mean AP is calculated as

(16) $$\mathrm{mAP}=\frac{1}{n}\sum_{k=1}^{n}AP_{k}$$

where *n* denotes the number of tumor classes, and ($AP_{k}$) is the Average Precision for class *k*.

Dice Similarity Coefficient (DSC)

To quantitatively assess the overlap between predicted and ground-truth tumor regions, we employ the Dice Similarity Coefficient (DSC), a widely used metric in medical image analysis. The Dice coefficient evaluates the agreement between two sets—typically the predicted segmentation and the reference annotation—by balancing both precision and recall. It is defined as

(17) $$\mathrm{DSC}=\frac{2\cdot TP}{2\cdot TP+FP+FN}$$

where *TP*, *FP*, and *FN* denote the number of true positives, false positives, and false negatives, respectively. The DSC ranges from 0 to 1, with 1 indicating perfect agreement. Compared to conventional accuracy, Dice is more robust in contexts of class imbalance, making it particularly well-suited for tumor detection tasks where positive instances are sparse and clinically significant. In this work, we report Dice scores for each tumor class to reflect the segmentation fidelity and model robustness. The use of Dice aligns with established practices in biomedical imaging and offers interpretable class-wise validation of model performance. This metric has a long-standing history in ecological and image analysis research [40].

Loss Components

The loss function guiding model optimization integrates three primary components: *llocalization loss* ($l_{\mathrm{box}}$), *lclassification loss* ($l_{\mathrm{cls}}$), and *ldistribution focal loss* ($l_{\mathrm{dfl}}$).

Localization Loss ($l_{\mathrm{box}}$): This term penalizes deviations between the predicted and ground-truth bounding box coordinates:

(18) $$l_{\mathrm{box}}=\lambda_{\mathrm{coord}}\sum_{i=0}^{S^{2}}\sum_{j=0}^{B}I_{ij}^{\mathrm{obj}}\left(2-w_{i}\times h_{i}\right)\left[{\left(x_{i}-{\hat{x}}_{i}\right)}^{2}+{\left(y_{i}-{\hat{y}}_{i}\right)}^{2}+{\left(w_{i}-{\hat{w}}_{i}\right)}^{2}+{\left(h_{i}-{\hat{h}}_{i}\right)}^{2}\right]$$

where

- *S* = number of grid cells in the image;
- *B* = number of bounding boxes per grid cell;
- $I_{ij}^{\mathrm{obj}}$ = indicator function (1 if the jth bounding box in cell *i* contains an object, 0 otherwise);
- $\left(x_{i},y_{i}\right)$ = predicted center coordinates of the bounding box;
- $\left({\hat{x}}_{i},{\hat{y}}_{i}\right)$ = ground-truth center coordinates;
- $\left(w_{i},h_{i}\right)$ = predicted width and height of the bounding box;
- $\left({\hat{w}}_{i},{\hat{h}}_{i}\right)$ = ground-truth width and height;
- $\lambda_{\mathrm{coord}}$ = weighting factor controlling the impact of localization loss.

The term $\left(2-w_{i}\times h_{i}\right)$ ensures that smaller bounding boxes receive a higher localization penalty, improving precision for small-object detection.

Classification Loss ($l_{\mathrm{cls}}$): This term measures the error between predicted class probabilities and the ground-truth class label:

(19) $$l_{\mathrm{cls}}=\lambda_{\mathrm{cls}}\sum_{i=0}^{S^{2}}\sum_{j=0}^{B}I_{ij}^{\mathrm{obj}}\sum_{c\in \mathrm{classes}}\left[p_{i}\left(c\right)\log\left({\hat{p}}_{i}\left(c\right)\right)\right]$$

where

- $p_{i}\left(c\right)$ = predicted probability for class *c* in cell *i*;
- ${\hat{p}}_{i}\left(c\right)$ = ground-truth probability for class *c* (1 for the correct class, 0 otherwise);
- $\lambda_{\mathrm{cls}}$ = weighting factor for the classification term;
- classes = set of all tumor categories.

These metrics were continuously monitored throughout the training and validation phases, facilitating effective model selection and enabling early stopping to prevent overfitting.

### 4.5. Loss Function Performance Assessment

**Figure 3:**
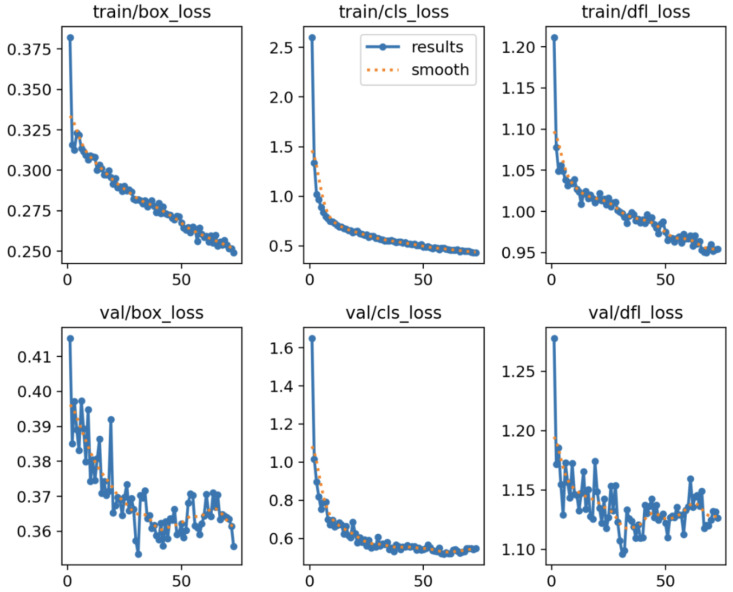
Assessing the performance of the loss function in the Brain Tumor Detection Model (BTDM).

Figure 3 presents the evolution of the loss components throughout the training and validation phases of the BTDM. Specifically, it displays the box regression loss (*box_loss*), the classification loss (*cls_loss*), and the distribution focal loss (*dfl_loss*) across 70 epochs. The top row shows training losses, while the bottom row corresponds to the validation set.

From the plots, we observe a consistent downward trend in all training loss components, indicating stable and effective learning. The training *box_loss* decreases from approximately 0.38 to 0.25, demonstrating improved localization accuracy of bounding boxes. Simultaneously, the *cls_loss* drops steeply from 2.6 to 0.45, reflecting a rapid enhancement in the model’s classification capability. The *dfl_loss*, crucial for high-precision object boundary estimation, falls from 1.21 to 0.95, confirming the model’s increasing precision in spatial distribution modeling.

The validation losses follow a similar trajectory, albeit with slightly higher variance, particularly for *dfl_loss*, which stabilizes around 1.12 after epoch 40. This coherence between training and validation losses suggests that the model generalizes well without signs of overfitting.

### 4.6. Recall and Precision Performance

The precision–recall (PR) curves provide a comparative evaluation of the BTDM and BTDS, developed using the proposed two-stage training architecture. In the first stage, the base model (BTDM) is trained on a large and diverse MRI dataset. It is selected based on its performance, specifically achieving a mean Average Precision (mAP) above 90%. In the second stage, this model is fine-tuned using transfer learning on a structurally similar but more specific brain tumor dataset to produce BTDS.

The PR curves assess detection performance across three tumor types—glioma, meningioma, and pituitary—during both training stages. The results reveal high precision and recall levels, confirming the effectiveness of the two-stage approach. Table 2 presents the class-wise detection performance of the proposed BTDM over five independent runs, reported as mean ± standard deviation for precision, recall, F1-score, and mAP@0.5. Overall, the model achieves excellent global performance, with an average F1-score of 0.899 ± 0.004 and an overall mAP@0.5 of 0.935 ± 0.004, indicating both high accuracy and consistency across runs.

A detailed interpretation of these results is provided as follows:

- Meningioma: Exhibits the highest detection performance across all metrics, with a nearly ideal precision–recall (PR) profile and an AP of 0.981. This suggests clear feature separability and low inter-class confusion for this tumor type.
- Pituitary: Achieves similarly strong results, with an AP of 0.962, indicating excellent localization and classification capabilities. The slightly lower recall compared to precision suggests a small proportion of missed detections.
- Glioma: While still robust, this class records the lowest AP (0.862) among the three, which may be attributed to higher intra-class variability, heterogeneous shapes, and more complex tumor boundaries.

These findings confirm that BTDM delivers consistent high-precision detection across diverse tumor types, with particularly strong performance on meningioma and pituitary adenomas, while maintaining competitive accuracy on the more challenging glioma category.

Table 3 reports the detection performance of BTDS, obtained via transfer learning from the base BTDM architecture. The model maintains strong global performance, achieving an overall mAP@0.5 of 0.909 ± 0.004, which, while marginally lower than BTDM, remains well above the 90% threshold. This consistency confirms the effectiveness and robustness of the proposed two-stage training and domain adaptation strategy.

The class-wise analysis reveals the following trends:

- Meningioma: Maintains near-optimal detection performance with an AP of 0.980, demonstrating excellent generalization and stability after transfer learning.
- Pituitary: Preserves high detection capability (AP = 0.911), with only a marginal drop relative to BTDM, reflecting robust adaptability to the target dataset.
- Glioma: Exhibits a more noticeable reduction in AP to 0.836, potentially due to domain shift effects and the complex morphological heterogeneity of this tumor type.

Across both BTDM and BTDS, meningioma and pituitary tumors achieve consistently high recall without compromising precision, reinforcing their reliability in clinical-grade detection scenarios. The slight degradation observed for glioma—particularly in precision at high recall thresholds—suggests the potential benefit of targeted data augmentation or class-specific model refinement.

Overall, this PR-based evaluation underscores the robustness and transferability of the BTDM architecture. The two-stage pipeline not only delivers high baseline accuracy but also adapts effectively to new datasets, making BTDS a strong candidate for integration into automated brain tumor detection systems and a valuable asset for translational deployment in medical imaging workflows.

### 4.7. Classification Performance Evaluation via Confusion Matrix

To comprehensively assess the classification performance of the proposed two-stage architecture, we examine the confusion matrices of the BTDM and BTDS. These matrices provide a fine-grained view of prediction accuracy across the three target tumor classes: glioma, meningioma, and pituitary. The results presented correspond to the runs whose global metrics (mAP@0.5, precision, and recall) were closest to the average across five repetitions. Comparable class-wise patterns were observed in other runs, underscoring the stability and reproducibility of the models’ predictive behavior.

Table 4 summarizes the confusion matrix-derived statistics, including true positives (TPs), false positives (FPs), false negatives (FNs), Dice coefficient, accuracy, and Matthews Correlation Coefficient (MCC), for each class.

- BTDM performance. The BTDM demonstrates strong classification ability across all tumor types, with particularly high performance for meningioma (Dice = 0.928) and pituitary tumors (Dice = 0.918). Glioma detection, while still robust (Dice = 0.818), is comparatively lower—likely reflecting the higher intra-class variability and complex morphological patterns characteristic of gliomas. Misclassifications include 68 false positives and 89 false negatives, suggesting a need for further refinement in boundary cases.
- BTDS performance after transfer learning. BTDS, fine-tuned from BTDM, sustains excellent performance for meningioma, achieving a Dice score of 0.984 with *zero* false negatives, highlighting its clinical reliability for this tumor type. For glioma, recall improves substantially (FN reduced from 89 to 13), albeit with a modest rise in false positives (from 68 to 26), yielding a slightly lower Dice of 0.802. Pituitary detection experiences a minor drop in Dice from 0.918 to 0.864, potentially due to reduced sample representation or domain shift in the fine-tuning dataset.
- Clinical interpretation and implications. Despite minor variations in per-class performance, both BTDM and BTDS achieve high accuracy and MCC values across tumor types, validating their suitability for clinical decision support. Importantly, the confusion matrix analysis reveals that most misclassifications occur near decision boundaries—e.g., gliomas misclassified as background—where human expertise remains essential. Rather than replacing clinicians, the proposed framework is designed to function as an *assistive* tool, flagging suspicious regions, prioritizing high-risk cases, and reducing the workload in high-throughput neuroimaging environments. This human-in-the-loop paradigm not only strengthens diagnostic accuracy but also ensures accountability, interpretability, and safe integration of AI into neuro-oncological workflows.

### 4.8. Post-Processing and Clinical Interpretation Pipeline

Following the initial detection performed by BTDS, a specialized post-processing pipeline is employed to refine the segmentation outputs and extract clinically actionable anatomical descriptors. Unlike standard YOLO-based segmentation—which typically yields coarse axis-aligned bounding boxes and lacks fine-grained delineation—our approach leverages a combination of contrast enhancement and morphological operations to achieve anatomically consistent high-precision tumor masks.

The pipeline starts by localizing the region of interest (ROI) using both the predicted bounding box and associated polygonal segmentation provided by the detection model. To enhance the visibility of low-contrast pathological structures—such as micro-lesions or peritumoral hyperintensities—the grayscale ROI undergoes histogram equalization. This preprocessing step is critical for amplifying subtle intensity variations within the tissue, thereby facilitating the identification of small spatially irregular tumor components often overlooked by conventional detectors.

Subsequent segmentation involves adaptive binarization through intensity thresholding, followed by morphological filtering (opening and closing) to eliminate high-frequency noise and to regularize the shape of the tumor region. The resulting mask is then reintegrated into the original spatial context and superimposed onto the source image, providing an accurate visual representation of the tumor extent. This mask serves as the basis for quantifying morphometric features—including tumor length, width, and surface area (cm^2^)—using a calibrated pixel-to-metric conversion factor.

**Figure 4:**
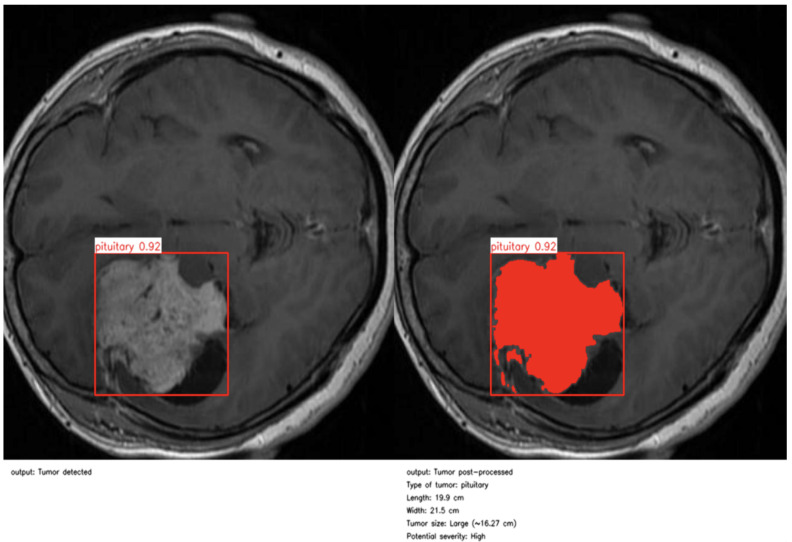
First example of post-processing and clinical report.

**Figure 5:**
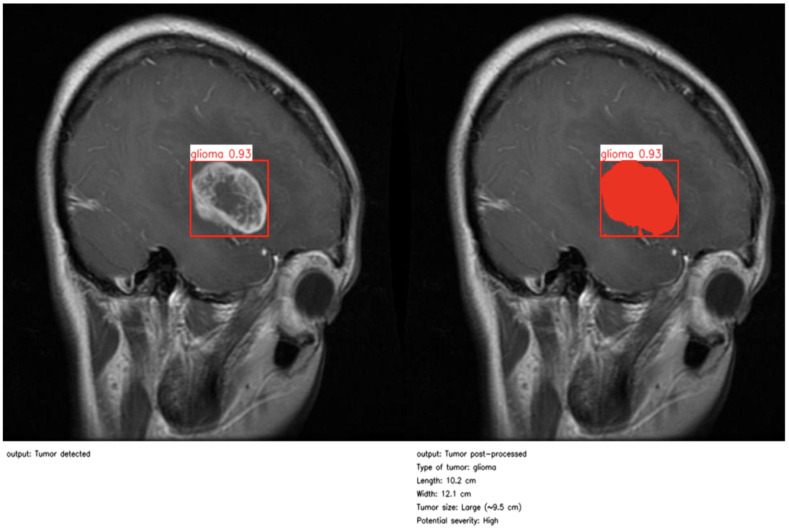
Second example of post-processing and clinical report.

As illustrated in Figure 4 and Figure 5, the proposed method enables precise post-detection analysis. For instance, a pituitary macroadenoma, detected with a high confidence score (0.92), exhibits a computed area of 16.27 cm^2^, corresponding to a large and clinically significant lesion. Similarly, a glioma lesion identified with 0.93 confidence yields dimensions of 10.2 × 12.1 cm and an estimated size of 9.5 cm^2^, indicative of aggressive growth potential. Notably, the contrast-enhanced pipeline enables the detection of peripheral hyperintense foci—small white patches within the ROI—which may correspond to early infiltrative zones or satellite tumor loci. These critical features are often indistinguishable in raw YOLO segmentation, highlighting the added diagnostic value of our approach.

This refined post-processing framework bridges the gap between object-level detection and pixel-wise pathological interpretation. By generating anatomically meaningful segmentation masks and quantitative descriptors, the system supports radiological assessment, enhances interpretability, and enables semi-automated report generation. As a result, our method functions not merely as a prediction tool but as an intelligent decision-support system aligned with real-world neuro-oncological workflows, capable of augmenting expert judgment while preserving clinical safety and transparency.

### 4.9. Comparative Analysis and Limitations

To contextualize the performance of the proposed two-stage YOLO-based framework for brain tumor detection and segmentation, we compared our results against recent state-of-the-art approaches (Table 5) and against a high-performing binary classification benchmark (Table 6).

Cengil et al. (2023) [39] investigated glioma and meningioma detection using a deep learning approach, reporting AP values of 0.786 and 0.926, respectively, with an overall mAP of 0.856. While their results are promising, the study is limited to binary classification and does not address additional tumor types, such as pituitary adenomas. In contrast, our BTDM achieves higher class-wise AP scores of 0.862 (glioma), 0.981 (meningioma), and 0.962 (pituitary), resulting in a mean mAP@0.5 of 0.935 across three classes—demonstrating stronger generalization and robustness.

Almufareh et al. (2024) [41] evaluated YOLOv5 and YOLOv7 on 827 labeled MRI scans containing meningioma, glioma, and pituitary adenomas. YOLOv5 achieved mean mAP@0.5 values of 0.947 (box) and 0.947 (mask), with per-class AP scores of 0.990 (meningioma), 0.978 (pituitary), and 0.872 (glioma). YOLOv7 produced slightly lower results, with mAP values of 0.940 (box) and 0.941 (mask) and AP scores of 0.985 (meningioma), 0.976 (pituitary), and 0.860 (glioma). While these results confirm the high localization capability of YOLO-based models, the evaluation was limited to a single dataset and did not investigate cross-dataset generalization. Our framework delivers comparable or superior AP scores for meningioma (0.981) and pituitary (0.962) while incorporating transfer learning to improve robustness to dataset variability.

From a classification perspective, Moldovanu et al. (2024) [42] combined the EfficientNetB0 architecture with a Support Vector Machine (SVM) classifier, achieving 99.5% test accuracy on a binary task distinguishing tumor-bearing from healthy brain MRI scans. Our BTDM and BTDS achieved test accuracies of 92.6% and 92.4%, respectively, on a substantially more challenging three-class problem involving gliomas, meningiomas, and pituitary tumors. It is well established that increasing the number of target classes—particularly in unbalanced medical datasets—tends to lower overall accuracy due to increased classification complexity and the added requirement to correctly localize as well as classify multiple pathological subtypes.

Unlike the binary setup in [42], our framework unifies object detection and pixel-wise segmentation, producing both class predictions and spatially precise lesion boundaries. This dual-output capability enhances clinical interpretability, enabling radiologists to assess lesion morphology, location, and extent directly from model predictions—providing high translational value for neuro-oncological diagnostics and treatment planning.

Despite its advantages, the proposed method has several limitations. The two-stage training pipeline, while improving fine-grained accuracy, increases training time and hyperparameter tuning complexity. Furthermore, although our dataset contains heterogeneous tumor types, its relatively modest size may limit the scalability of the model to diverse clinical environments. Future work will focus on incorporating larger multi-institutional datasets (e.g., BraTS), leveraging domain adaptation techniques, and exploring self-supervised learning strategies to enhance model generalization and reduce reliance on extensive manual annotations.

## 5. Conclusions

In this article, we presented a clinically oriented deep learning pipeline for the detection, classification, and segmentation of brain tumors from MRI scans, designed to maximize both predictive accuracy and clinical interpretability. The proposed two-stage architecture, comprising the Brain Tumor Detection Model (BTDM) and Brain Tumor Detection and Segmentation (BTDS), leverages transfer learning to maintain high performance across diverse datasets, even in low-resource settings. The experimental results demonstrate the robustness of the approach, with the BTDM achieving an mAP@0.5 of 0.935 and BTDS retaining strong generalization with a score of 0.909, as confirmed by precision–recall curves and confusion matrix analysis.

A distinctive feature of this framework lies in its post-processing module, which transforms raw model outputs into clinically relevant indicators such as tumor size, shape, and severity level. This enhances transparency, facilitates expert interpretation, and reinforces the role of the system as an assistive decision-support tool rather than a replacement for medical expertise.

Looking ahead, several research directions are envisioned to extend the impact and applicability of this work:

- Three-dimensional volumetric analysis to improve spatial consistency across MRI slices.
- Temporal tracking for longitudinal monitoring of tumor progression.
- Uncertainty estimation mechanisms to automatically flag ambiguous or borderline cases for expert review.
- Multi-modal integration combining MRI with other imaging modalities (e.g., CT and PET) for richer diagnostic context.
- Continual learning strategies to enable incremental adaptation of the model as new data become available, avoiding catastrophic forgetting and ensuring sustained accuracy in dynamic clinical environments. Recent advances [43,44] illustrate the potential of such approaches and provide a foundation for their adaptation to neuro-oncological imaging.

Finally, prospective validation through clinical collaborations will be pursued to rigorously assess the system’s real-world utility. By aligning methodological innovation with clinical needs, this framework lays the groundwork for next-generation, explainable, and deployable AI systems in neuro-oncology diagnostics.

## Figures and Tables

**Table 2:** Detection performance of the BTDM over five independent runs (mean ± std).

Class	Precision (mean ± std)	Recall (mean ± std)	F1-Score (mean ± std)	mAP@0.5 (mean ± std)
Glioma	0.838 ± 0.006	0.798 ± 0.008	0.817 ± 0.005	0.862 ± 0.007
Meningioma	0.966 ± 0.004	0.959 ± 0.005	0.962 ± 0.003	0.981 ± 0.003
Pituitary	0.948 ± 0.005	0.891 ± 0.007	0.919 ± 0.004	0.962 ± 0.004
All classes (mean)	–	–	0.899 ± 0.004	0.935 ± 0.004

**Table 3:** Detection performance of BTDS over five independent runs (mean ± std).

Class	Precision (mean ± std)	Recall (mean ± std)	F1-Score (mean ± std)	mAP@0.5 (mean ± std)
Glioma	0.752 ± 0.007	0.859 ± 0.006	0.802 ± 0.005	0.836 ± 0.005
Meningioma	0.968 ± 0.004	1.000 ± 0.000	0.984 ± 0.002	0.980 ± 0.003
Pituitary	0.912 ± 0.006	0.820 ± 0.008	0.863 ± 0.005	0.911 ± 0.005
All classes (mean)	–	–	0.883 ± 0.004	0.909 ± 0.004

**Table 4:** Comparison of classification metrics between BTDM and BTDS (TP, FP, FN, Dice, accuracy, and MCC).

Class	BTDM						BTDS					
	TP	FP	FN	Dice	Accuracy	MCC	TP	FP	FN	Dice	Accuracy	MCC
Glioma	352	68	89	0.818	0.842	0.684	79	26	13	0.802	0.857	0.714
Meningioma	259	9	11	0.928	0.981	0.939	92	3	0	0.984	0.996	0.983
Pituitary	253	14	31	0.918	0.955	0.910	73	7	16	0.864	0.918	0.841
Mean	–	–	–	0.888	0.926	0.844	–	–	–	0.883	0.924	0.846

**Table 5:** Comparative performance of recent brain tumor detection methods (mAP@0.5).

Study/Model	Glioma	Meningioma	Pituitary	mAP@0.5
Cengil et al. (2023) [39]	0.786	0.926	–	0.856
Almufareh et al. (2024)–YOLOv5 [41]	0.872	0.990	0.978	0.947
Almufareh et al. (2024)–YOLOv7 [41]	0.860	0.985	0.976	0.940
Proposed method (BTDM)	0.862	0.981	0.962	0.935

**Table 6:** Comparison of test accuracy: proposed models vs. EfficientNetB0–SVM (reference).

Model	Task	Accuracy (%)
EfficientNetB0 + SVM [42]	Binary tumor vs. healthy classification	99.5
BTDM	Multi-class tumor detection (glioma, meningioma, pituitary)	92.6
BTDS	Multi-class tumor detection (glioma, meningioma, pituitary)	92.4

## Data Availability

The datasets analyzed during the current study are publicly available. Three datasets were used to evaluate the performance of the methodology: Dataset 1: https://universe.roboflow.com/final-eyj5r/brain-mri-pqpcm, accessed on 20 May 2025, Roboflow Dataset 2: https://universe.roboflow.com/my-thesis-zy14w/mergechngbvj, accessed on 20 May 2025, and Roboflow Dataset 3: https://universe.roboflow.com/lll-czafp/project2-blspd/dataset/2, accessed on 20 May 2025.

## Abbreviations

The following abbreviations are used in this manuscript:

BTDM	Brain Tumor Detection Model
BTDS	Brain Tumor Detection and Segmentation
MRI	Magnetic Resonance Imaging

## References

[1] American Cancer Society Key Statistics for Brain and Spinal Cord Tumors. https://www.cancer.org/cancer/types/brain-spinal-cord-tumors-adults/about/key-statistics.html.

[2] Lapointe S., Perry A., Butowski N.A. (2018). Primary brain tumours in adults. Lancet.

[3] Zacharaki E.I., Wang S., Chawla S., Yoo D.S., Wolf R., Melhem E.R., Davatzikos C. (2009). Classification of brain tumor type and grade using MRI texture and shape in a machine learning scheme. Magn. Reson. Med..

[4] Liu J., Li M., Wang J., Wu F., Liu T., Pan Y. (2014). A survey of MRI-based brain tumor segmentation methods. Tsinghua Sci. Technol..

[5] Abiwinanda N., Hanif M., Hesaputra S.T., Handayani A., Mengko T.R. (2019). Brain tumor classification using convolutional neural network. Proceedings of the World Congress on Medical Physics and Biomedical Engineering.

[6] Amin J., Sharif M., Haldorai A., Yasmin M., Nayak R.S. (2022). Brain tumor detection and classification using machine learning: A comprehensive survey. Complex Intell. Syst..

[7] Watts J., Box G., Galvin A., Brotchie P., Trost N., Sutherland T. (2014). Magnetic resonance imaging of meningiomas: A pictorial review. Insights Into Imaging.

[8] Swati Z.N.K., Zhao Q., Kabir M., Ali F., Ali Z., Ahmed S., Lu J. (2019). Brain tumor classification for MR images using transfer learning and fine-tuning. Comput. Med. Imaging Graph..

[9] Litjens G., Kooi T., Bejnordi B.E., Setio A.A.A., Ciompi F., Ghafoorian M., van der Laak J.A.W.M., van Ginneken B., Sánchez C.I. (2017). A survey on deep learning in medical image analysis. Med. Image Anal..

[10] Lundervold A.S., Lundervold A. (2019). An overview of deep learning in medical imaging focusing on MRI. Z. Med. Phys..

[11] Sharma R.M. (2025). Artificial intelligence in medical image analysis and molecular diagnostics: Recent advances and applications. J. Med. Artif. Intell..

[12] Izhar A., Idris N., Japar N. (2025). Medical radiology report generation: A systematic review of current deep learning methods, trends, and future directions. Artif. Intell. Med..

[13] Obuchowicz R., Lasek J., Wodziński M., Piórkowski A., Strzelecki M., Nurzynska K. (2025). Artificial Intelligence-Empowered Radiology—Current Status and Critical Review. Diagnostics.

[14] Chau M., Vu H., Debnath T., Rahman M. (2025). A scoping review of automatic and semi-automatic MRI segmentation in human brain imaging. Radiography.

[15] Zhao Z.Q., Zheng P., Xu S., Wu X. (2019). Object detection with deep learning: A review. IEEE Trans. Neural Netw. Learn. Syst..

[16] Imran M., Anwar H., Tufail M., Khan A., Khan M., Ramli D.A. (2023). Image-based automatic energy meter reading using deep learning. Comput. Mater. Contin..

[17] Alwakid G., Gouda W., Humayun M. (2023). Deep learning-based prediction of diabetic retinopathy using CLAHE and ESRGAN for enhancement. Healthcare.

[18] Liu W., Anguelov D., Erhan D., Szegedy C., Reed S., Fu C.Y., Berg A.C. (2016). SSD: Single shot MultiBox detector. Computer Vision—ECCV.

[19] Girshick R., Donahue J., Darrell T., Malik J. Rich feature hierarchies for accurate object detection and semantic segmentation. Proceedings of the IEEE Conference on Computer Vision and Pattern Recognition.

[20] Ren S., He K., Girshick R., Sun J. (2017). Faster R-CNN: Towards real-time object detection with region proposal networks. IEEE Trans. Pattern Anal. Mach. Intell..

[21] Ramachandran S., George J., Skaria S., Mori K., Petrick N. Using YOLO based deep learning network for real-time detection and localization of lung nodules from low dose CT scans. Proceedings of the SPIE Medical Imaging.

[22] Dorfner F.J., Patel J.B., Kalpathy-Cramer J., Gerstner E.R., Bridge C.P. (2025). A review of deep learning for brain tumor analysis in MRI. NPJ Precis. Oncol..

[23] Hossain A., Islam M.T., Almutairi A.F. (2022). A deep learning model to classify and detect brain abnormalities in portable microwave-based imaging system. Sci. Rep..

[24] Shelatkar T., Bansal U. (2023). Diagnosis of brain tumor using lightweight deep learning model with fine-tuning approach. Machine Learning and Computational Intelligence Techniques for Data Engineering.

[25] Talukder M.A., Islam M.M., Uddin M.A., Akhter A., Pramanik M.A.J., Aryal S., Almoyad M.A.A., Hasan K.F., Moni M.A. (2023). An efficient deep learning model to categorize brain tumor using reconstruction and fine-tuning. Expert Syst. Appl..

[26] Salman L.A., Hashim A.T., Hasan A.M. (2022). Automated brain tumor detection of MRI image based on hybrid image processing techniques. Telkomnika.

[27] Havaei M., Davy A., Warde-Farley D., Biard A., Courville A., Bengio Y., Pal C., Jodoin P.-M., Larochelle H. (2017). Brain tumor segmentation with deep neural networks. Med. Image Anal..

[28] Ben Brahim S., Dardouri S., Bouallegue R. (2024). Brain tumor detection using a deep CNN model. Appl. Comput. Intell. Soft Comput..

[29] Adhikari B., Kulung P., Bohaju J., Poudel L.K., Raymond C., Zhang D., Anazodo U.C., Khanal B., Shakya M. (2024). Parameter-efficient fine-tuning for improved convolutional baseline for brain tumor segmentation in Sub-Saharan Africa adult glioma dataset. Preprint or journal information to be updated if available. arXiv.

[30] Mabray M.C., Barajas R.F., Cha S. (2015). Modern brain tumor imaging. Brain Tumor Res. Treat..

[31] Cengil E., Çınar A. (2021). Poisonous mushroom detection using YOLOv5. Turk. J. Sci. Technol..

[32] Jocher G., Derrenger P., Munawar M.R. Home–Ultralytics YOLO Docs. https://docs.ultralytics.com/.

[33] Jocher G., Derrenger P. yolo11.yaml. https://github.com/ultralytics/ultralytics/blob/main/ultralytics/cfg/models/11/yolo11.yaml.

[34] Hidayatullah P., Syakrani N., Sholahuddin M.R., Gelar T., Tubagus R. (2025). YOLOv8 to YOLO11: A Comprehensive Architecture In-depth Comparative Review. arXiv.

[35] Bochkovskiy A., Wang C.Y., Liao H.Y.M. (2020). Yolov4: Optimal speed and accuracy of object detection. arXiv.

[36] Kaur P., Khehra B.S., Mavi E.B.S. Data augmentation for object detection: A review. Proceedings of the 2021 IEEE International Midwest Symposium on Circuits and Systems (MWSCAS).

[37] Zhang H., Zhang S., Zou R. (2024). Select-Mosaic: Data Augmentation Method for Dense Small Object Scenes. arXiv.

[38] Alruwaili M., Atta M.N., Siddiqi M.H., Khan A., Khan A., Alhwaiti Y., Alanazi S. (2023). Deep learning-based YOLO models for the detection of people with disabilities. IEEE Access.

[39] Almufareh M.F., Imran M., Khan A., Humayun M., Asim M. (2024). Automated brain tumor segmentation and classification in MRI using YOLO-based deep learning. IEEE Access.

[40] Shamir R.R., Duchin Y., Kim J., Sapiro G., Harel N. (2019). Continuous Dice coefficient: A method for evaluating probabilistic segmentations. arXiv.

[41] Cengil E., Eroğlu Y., Çınar A., Yıldırım M. (2023). Detection and Localization of Glioma and Meningioma Tumors in Brain MR Images using Deep Learning. Sak. Univ. J. Sci..

[42] Moldovanu S., Tăbăcaru G., Barbu M. (2024). Convolutional neural network–machine learning model: Hybrid model for meningioma tumour and healthy brain classification. J. Imaging.

[43] Ren X., Qin Y., Li B., Wang B., Yi X., Jia L. (2024). A core space gradient projection-based continual learning framework for remaining useful life prediction of machinery under variable operating conditions. Reliab. Eng. Syst. Saf..

[44] Wang T., Guo D., Sun X.-M. (2023). Contrastive generative replay method of remaining useful life prediction for rolling bearings. IEEE Sens. J..

